# Transcriptomic comparative study between canalicular adenoma and *HMGA2* :: *WIF1* canalicular‐like pleomorphic adenoma

**DOI:** 10.1002/path.70070

**Published:** 2026-04-29

**Authors:** Ziyad Alsugair, Anne Champagnac, Maxime Fieux, Pierre Philouze, Philippe Ceruse, Brice Thamphya, Daniel Pissaloux, Franck Tirode, Nazim Benzerdjeb

**Affiliations:** ^1^ Service d'Anatomie et Cytologie Pathologiques, Institut de Pathologie Multisite, Hospices Civils de Lyon, CHU Lyon Sud Pierre‐Bénite France; ^2^ Département de Biopathologie Centre Leon Berard Lyon France; ^3^ Service of Oto‐Rhino‐Laryngology, CHU Sud, Hospices Civils de Lyon Pierre Bénite France; ^4^ Université Claude Bernard Lyon 1 Lyon France; ^5^ Service d'Oto‐Rhino‐Laryngologie et Chirurgie Cervico‐Faciale, Hôpital La Croix Rousse, Hospices Civils de Lyon Lyon France; ^6^ Team Genetics, Epigenetics and Biology of Sarcomas, Centre de Recherche en Cancérologie de Lyon, INSERM U1052 – CNRS UMR5286, Centre Léon Bérard Université Claude Bernard Lyon 1 Lyon France; ^7^ EMR3738, CICLY Pierre‐Bénite France

**Keywords:** *HMGA2*::*WIF1*, canalicular adenoma, canalicular‐like pleomorphic adenoma, transcriptomic analysis, salivary gland tumor, head and neck pathology, classification, biomarkers, SOX2

## Abstract

Canalicular adenoma (CA) is a benign salivary gland tumor predominantly affecting the upper lip and is characterized by monomorphic epithelial cells arranged in branching cords within a loose and vascularized stroma. Unlike pleomorphic adenoma (PA), CA lacks a chondroid matrix and exhibits specific histological features, such as high cuboidal to columnar cells arranged in one to two cell layers. A recently identified PA subtype, named ‘*HMGA2*‐canalicular‐like PA’, mimics the morphology of CA but affects major salivary glands, and harbors the *HMGA2*::*WIF1* fusion. Immunohistochemical markers including SOX10 positivity and p40/p63 negativity are shared between these entities, raising questions about their molecular differences. Therefore, the current study aimed to extensively compare the molecular profiles of CAs and *HMGA2*‐canalicular‐like PAs to better understand the mechanisms underlying their oncogenesis. In the present study, CAs and the *HMGA2*‐canalicular‐like PAs mostly shared similar histology and immunostaining, while the clinical presentations regarding tumor site and transcriptomic profiles were different. A novel finding was the overexpression of SOX2 in patients diagnosed with CA, in comparison to those diagnosed with *HMGA2*‐canalicular‐like PA. The CAs displayed significantly higher enrichment of hallmarks for Hedgehog signaling, IL2/STAT5 signaling, and apical surface, and significantly lower enrichment of hallmarks for apoptosis and mitotic spindle. Additionally, Gene Ontology enrichment analysis displayed significant enrichment of biological process for CAs in comparison to *HMGA2*‐canalicular‐like PAs for myofibril assembly, muscle filament sliding, actin–myosin filament sliding, and sarcomere organization. CAs and *HMGA2*‐canalicular‐like PAs exhibited similar histology and immunostaining but differed significantly in tumor site and transcriptomic profiles. The latter revealed significant activation of muscle‐related transcriptional pathways, and overexpression of SOX2 in CAs. © 2026 The Author(s). *The Journal of Pathology* published by John Wiley & Sons Ltd on behalf of The Pathological Society of Great Britain and Ireland.

## Introduction

Canalicular adenomas (CAs) are defined as benign salivary gland neoplasms of monomorphic epithelial cells [1]. These tumors are characterized by uniform high cuboidal to columnar cells organized in one to two cell layers, forming anastomoses, branching cords, rows, or canaliculi that align parallel. These structures are supported by a loose and myxoid stroma that is highly vascularized and often accompanied by sclerosing to fibrillar collagen deposition and areas of luminal hemorrhage. Notably, these tumors lack a chondroid matrix. CAs predominantly occur in the upper lip (80%, followed by the buccal mucosa, lower lip, hard palate, and soft palate) [2]. However, a recently identified subtype of pleomorphic adenoma (PA), named ‘canalicular adenoma‐like’, exhibits a morphological pattern similar to that of CA and frequently harbors the *HMGA2*::*WIF1* gene fusion (*HMGA2*‐canalicular‐like PA) [3, 4]. *HMGA2*::*WIF1*‐positive PAs occur across subtypes. Although they may be overrepresented among recurrent cases in selected series, available data are limited and suggest higher aggressiveness or malignant transformation rates compared with conventional pleomorphic adenoma [4, 5]. However, immunohistochemical similarities have also been reported, including negativity for p40 and p63 as well as positivity for SOX10 and S100. These findings raise questions regarding the molecular mechanisms underlying the immunohistochemical and histological similarities, as well as the differences in location (major versus minor salivary glands). Therefore, the present study aimed to extensively compare the molecular profiles of CAs and *HMGA2*‐canalicular‐like PAs to better understand the mechanisms underlying their oncogenesis.

## Materials and methods

### Ethical approval

The study was approved by the institutional review board of the Hospices Civils de Lyon (22‐5097) and was conducted in accordance with ethical guidelines (https://doi.org/10.1016/j.accpm.2018.10.013).

### Case selection

In the present study, cases of CA and PA harboring *HMGA2*::*WIF1* fusion were retrospectively selected by pathologists from two French pathology departments. PA cases harboring *HMGA2*::*WIF1* fusion were classified based on their histological morphology into two groups: the first group included PAs exhibiting a purely monomorphic appearance without a conventional component, designated as ‘canalicular‐like pleomorphic adenoma’ (*HMGA2‐*canalicular‐like PA); the second group comprised hybrid PAs displaying both conventional and monomorphic areas, designated as ‘conventional PA’ (*HMGA2‐*PA) [4]. Each department provided samples of the selected cases that were reviewed, and reclassified when required, by two pathologists (NB, ZA).

### Immunohistochemistry

Tissue specimens were fixed in 10% formaldehyde, embedded in paraffin, and processed routinely. The diagnosis of each tumor was based on light microscopic examination of conventionally prepared sections stained with hematoxylin–phloxine–saffron (HPS) or hematoxylin–eosin–saffron (HES) according to the last World Health Organization (WHO) classification [1]. Immunohistochemistry (IHC) was performed on 4‐μm sections cut from paraffin blocks using a fully automated system (Benchmark XT System; Ventana Medical Systems Inc., Tucson, AZ, USA) and the following antibodies: CK7 (clone OV‐TL, 1:1,000; Biogenex, Fremont, CA, USA), p40 (ΔNp63, polyclonal, 1:100; Zytomed, Berlin, Germany), p63 (clone SSI6, 1:100; DCS Innovative Diagnostik‐Systeme, Hamburg, Germany), S100 protein (polyclonal, 1:2,500; Dako, Glostrup, Denmark), and SOX10 (polyclonal, 1:25; DCS Innovative Diagnostik‐Systeme). The HMGA2 IHC was performed using a monoclonal rabbit antibody (clone EP398, 1:600; Epitomics, Burlingame, CA, USA; and clone HMGA2‐P1, 1:200; Biocheck, Foster City, CA, USA). Only unequivocal nuclear staining with the HMGA2 antibodies was considered. The SOX2 IHC was performed using a monoclonal rabbit antibody (clone D6D9, 1:200; Cell Signaling Technology, Danvers, MA, USA). Each case was reviewed by two pathologists (NB, ZA).

### Whole‐exome capture RNA sequencing

Exome‐based RNA capture sequencing was performed on formalin‐fixed, paraffin‐embedded samples; the data were further analyzed to detect fusion genes and small nucleotide variations, and to compare expression profiles with more than 8,000 other samples using clustering methods. The molecular basis of the technique, the technical protocol, and the bioinformatic algorithms used have been previously described [6]. This analysis was performed on a cohort of five cases of CA, eight cases of *HMGA2*‐canalicular‐like PA, nine cases of *HMGA2*‐PA, 11 cases of *PLAG1*‐rearranged pleomorphic adenoma (*PLAG1*‐PA), eight cases of carcinoma ex‐pleomorphic adenoma (CXPA), ten cases of polymorphous adenocarcinoma, 11 cases of basal cell adenoma, one case of basal cell adenocarcinoma, eight cases of acinic cell carcinoma of the salivary glands, four cases of salivary duct carcinoma of the salivary glands, two cases of sinonasal undifferentiated carcinoma, ten cases of oncocytoma, and 20 cases of adenoid cystic carcinoma of the salivary glands.

### Statistical analyses

Distributions of expression values within each group were assessed for normality using the Kolmogorov–Smirnov test. Pairwise comparisons of expression profiles were conducted using Fisher's exact test, followed by *t*‐tests with Bonferroni correction for multiple testing. Differentially expressed genes were identified using the Python package PyDESeq2 (https://github.com/scverse/PyDESeq2), as well as the false discovery rate (FDR). Unsupervised clustering analyses were performed on all genes without selection. A clustering tree was created using the Ward method, and distance evaluation was carried out using Pearson's and Spearman's correlation methods, represented as a dendrogram using the scikit‐learn package in Python (https://github.com/scikit-learn/scikit-learn). Uniform manifold approximation and projection (UMAP) was generated using the umap‐learn package (https://github.com/lmcinnes/umap). *SOX2* and *HMGA2* expression analyses among groups were performed using *t*‐tests. Violin plots were created with the seaborn package (https://github.com/mwaskom/seaborn). The gene set enrichment analysis (GSEA) methods used are based on enrichment analysis of MSigDB [7] and Gene Ontology (GO) [8]. These analyses were performed using the GSEApy package (https://github.com/zqfang/GSEApy). A hybrid radar‐violin plot with a stellar design, named ‘stellar violin radar plot’ was created in Python, based on the violin plot method and adapted by our team (NB and FT) [9]. The stellar violin radar plot was used to summarize the analysis of the hallmark signatures from the Human Molecular Signatures Database (https://www.gsea-msigdb.org/gsea/msigdb) to compare the CAs and *HMGA2*‐canalicular‐like PAs. A *p* value less than 0.05 was considered statistically significant.

## Results

### Clinical features

The study cohort was composed of two groups: five patients diagnosed as having CA and eight patients diagnosed as having *HMGA2*‐canalicular‐like PA. In the group of patients diagnosed with CA, which occurred in the oral cavity (5/5; 100.0%), the mean tumor size was 12.6 mm (range 7–30 mm), most of the patients were females (4/5; 80.0%), and the mean age was 66.6 years (range 61–76 years; Table 1). The patients treated for CA did not experience any recurrence. Regarding the group of patients treated for *HMGA2*‐canalicular‐like PA, the tumor occurred in the parotid (7/8, 87.5%) and submandibular glands (1/8, 12.5%), the mean size of the tumor was 36.6 mm (range 12–80 mm), most of the patients were males (6/8, 75.0%), and the mean age was 71.5 years (range 57–83 years; Table 1). Among them, three patients (3/8, 37.5%) experienced a recurrence at 18, 23, and 240 months, respectively.

**Table 1:** Clinicopathological and molecular features.

Histological type	Age (years)	Sex	Site	Size (mm)	p63/p40 IHC	RNA‐seq
CA	68	F	Upper lip	10	p63−/p40−	No fusion
CA	61	F	Upper lip	8	p63−/p40−	No fusion
CA	76	F	Oral cavity	30	p63−/p40−	No fusion
CA	68	F	Lip	7	p63−/p40−	No fusion
CA	60	M	Upper lip	8	p63−/p40−	No fusion
*HMGA2*‐canalicular‐like PA	63	F	Parotid	12	p63−/p40−	*HMGA2*::*WIF1*
*HMGA2*‐canalicular‐like PA	72	F	Parotid	22	p63−/p40−	*HMGA2*::*WIF1*
*HMGA2*‐canalicular‐like PA	57	M	Parotid	25	p63−/p40−	*HMGA2*::*WIF1*
*HMGA2*‐canalicular‐like PA	67	M	Parotid	38	p63−/p40−	*HMGA2*::*WIF1*
*HMGA2*‐canalicular‐like PA	77	F	L parotid	21	p63−/p40−	*HMGA2*::*WIF1*
*HMGA2*‐canalicular‐like PA	80	M	R submandibular gland	80	p63−/p40−	*HMGA2*::*WIF1*
*HMGA2*‐canalicular‐like PA	73	M	R parotid	35	p63−/p40−	*HMGA2*::*WIF1*
*HMGA2*‐canalicular‐like PA	83	M	R parotid	60	p63−/p40−	*HMGA2*::*WIF1*

CA, canalicular adenoma; F, female; IHC, immunohistochemistry; L, left; M, male; PA, pleomorphic adenoma; R, right; RNA‐seq, ribonucleic acid sequencing; −, negative; +, positive.

### Pathological and immunohistochemical features

**Figure 1:**
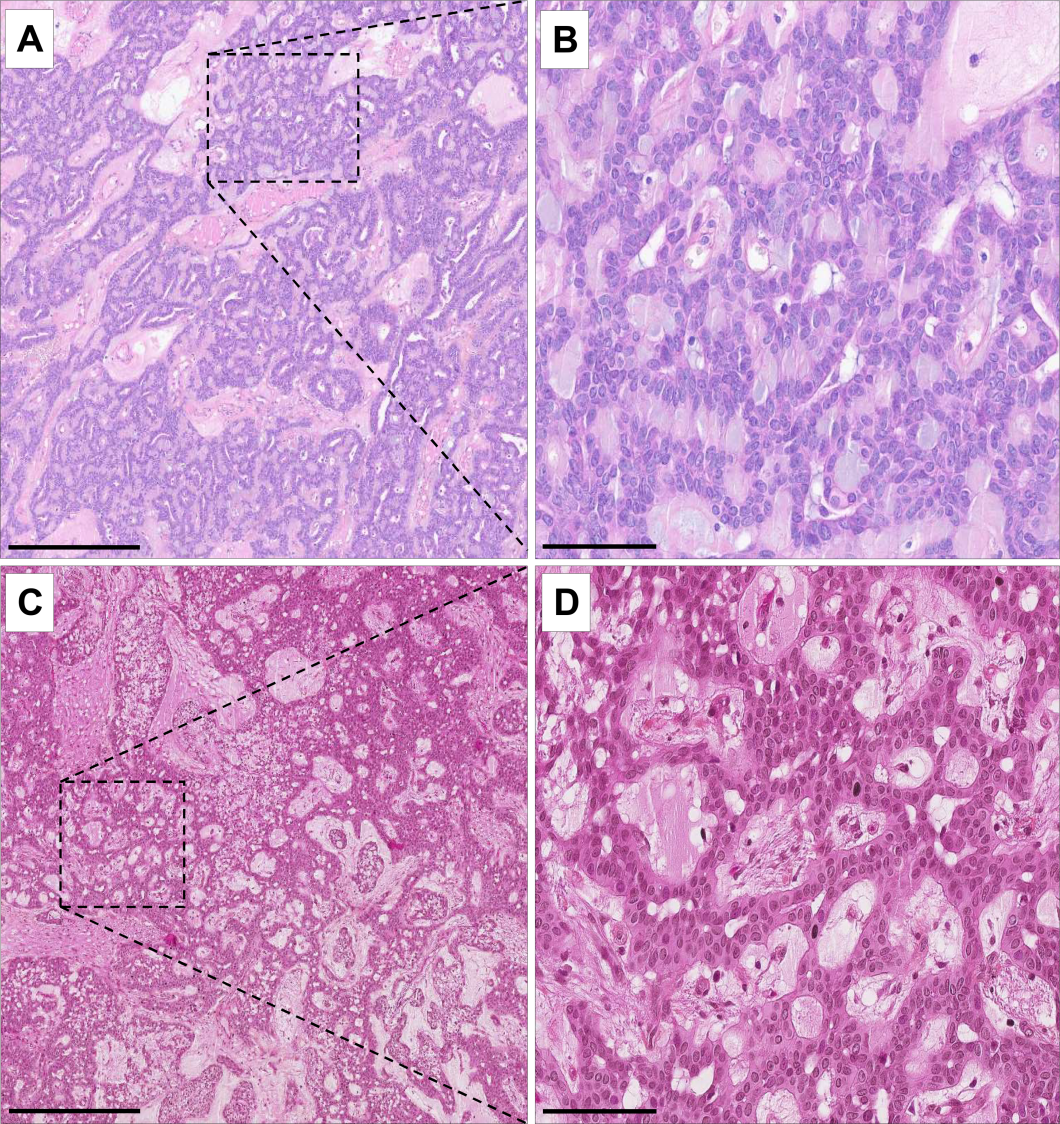
Histological features of canalicular adenomas (CAs) and *HMGA2*‐canalicular‐like pleomorphic adenomas (PAs). Mostly, the tumors were tightly packed and formed irregular anastomoses and branching patterns in (A) CAs [hematoxylin–eosin–saffron (HES), ×4; scale bar, 500 μm] and (C) *HMGA2*‐canalicular‐like PAs [hematoxylin–phloxine–saffron (HPS), ×4; scale bar, 500 μm]. The tumor cells were consistently monotonous, ranging from high cuboidal to columnar epithelial cells and arranged in cords, columns, trabeculae, or canaliculi for (B) CAs (HES, ×10; scale bar, 100 μm) and (D) *HMGA2*‐canalicular‐like PAs (HPS, ×10; scale bar, 100 μm).

Histologically, the tumors were well defined. Whether classified as CAs or *HMGA2*‐canalicular‐like PAs, the tumor cells were consistently monotonous, ranging from high cuboidal to columnar epithelial cells. These cells were tightly packed and formed irregular anastomoses and branching patterns (Figure 1A,C). The tumor cells were arranged in budding strands, cords, columns, trabeculae, or canaliculi, typically of at least two cells thick. Within these interconnected strands, trapped tubules, small acinar structures, intercalated duct‐like elements, or microcystic gland‐like spaces were present. In some areas, a solid, confluent growth pattern was observed, consisting of compressed trabeculae and small intercalated duct‐like glands. The nuclei were monomorphic, displaying minimal or no atypia, while the cytoplasm was moderately eosinophilic and finely granular (Figure 1B,D). None of the tumors exhibited significant cytologic atypia, increased mitotic activity, necrosis, or invasion through the capsule. The immunohistochemistry revealed consistent positive expression of CK7, SOX10, and S100. The p63−/p40− profile was observed in all CAs and *HMGA2*‐canalicular‐like PAs. HMGA2 immunohistochemistry showed intense and diffuse homogeneous nuclear reactivity in all cases of *HMGA2*‐canalicular‐like PA and was negative in all CAs.

### Molecular analysis

**Figure 2:**
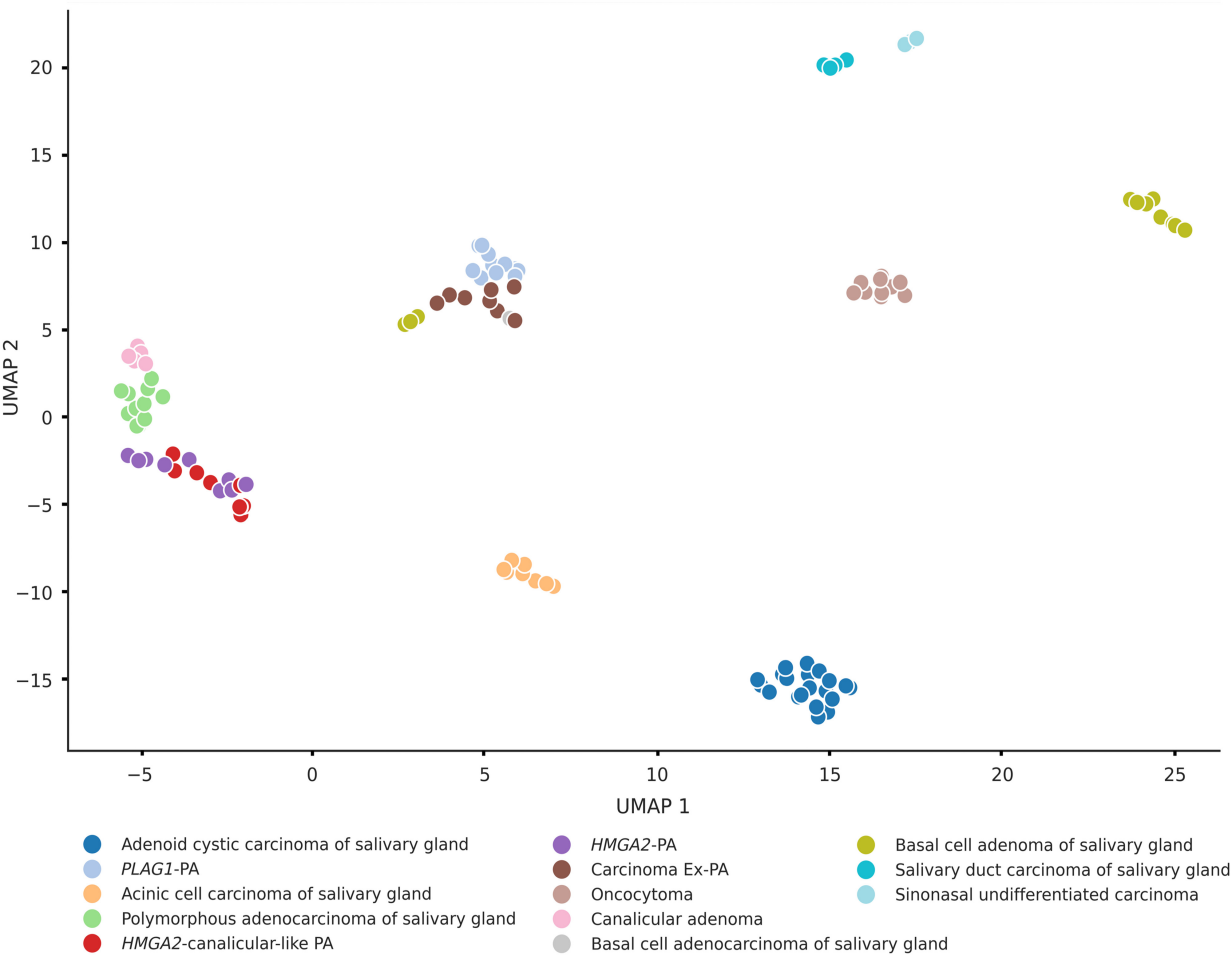
Transcriptome‐based clustering analysis. After nonlinear dimensionality reduction [uniform manifold approximation and projection (UMAP)], the canalicular adenomas (CAs) clustered together, close to *HMGA2*‐canalicular‐like pleomorphic adenomas (PAs), *HMGA2*‐PAs, and polymorphous adenocarcinomas, and were separate from the other cases.

Clustering analysis using gene expression profiling was performed by UMAP, which showed that the five CAs clustered together and were close to the *HMGA2*‐canalicular‐like PAs, the *HMGA2*‐PAs, and the polymorphous adenocarcinomas. The five CAs constituted a separate group from all other neoplasms (Figure 2).

**Figure 3:**
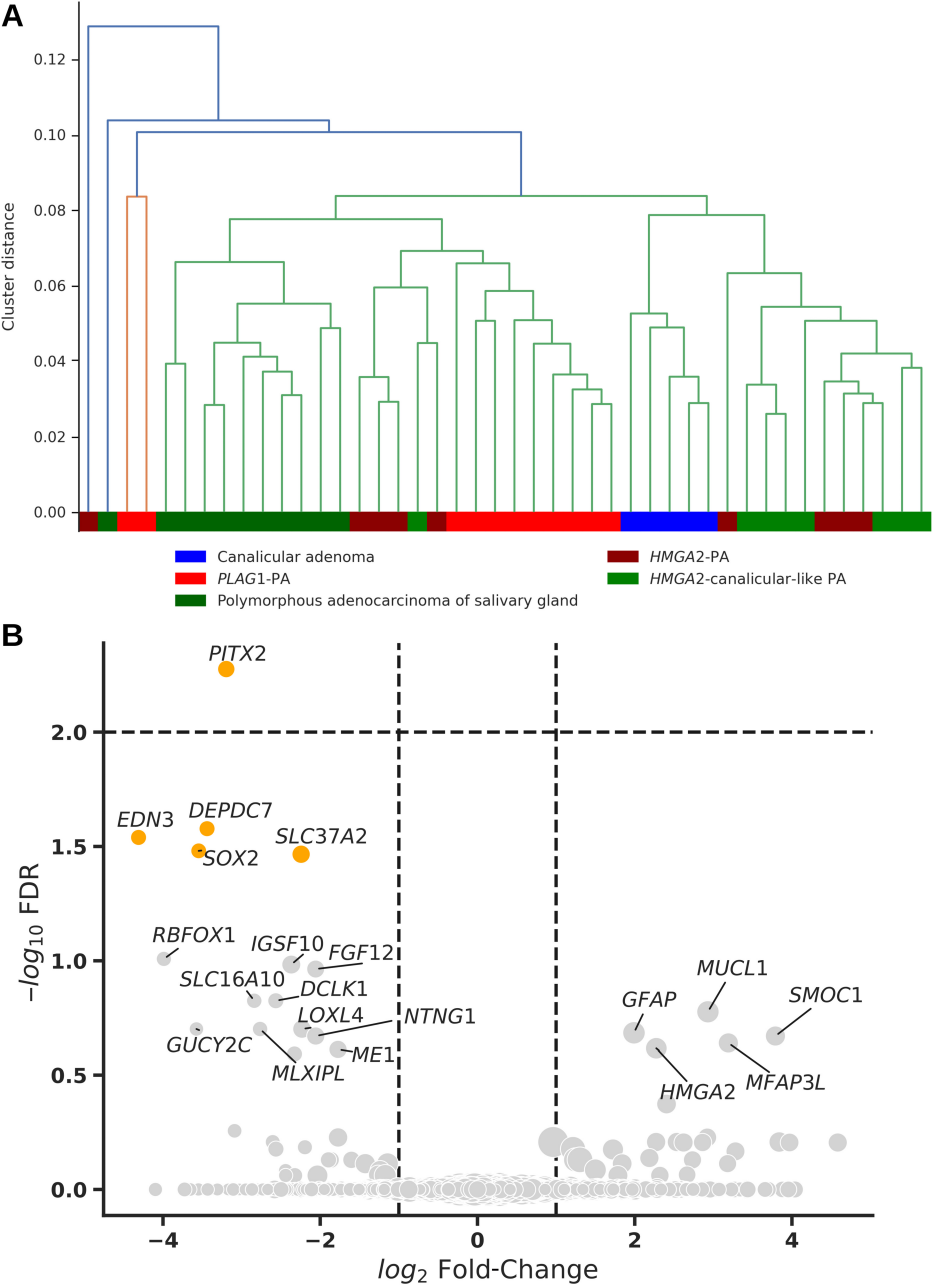
Transcriptome analysis of distinctiveness between canalicular adenomas (CAs) and *HMGA2*‐canalicular‐like pleomorphic adenomas (PAs). (A) The hierarchical clustering dendrogram shows that the CAs are significantly closer to the *HMGA2*‐canalicular‐like PAs and *HMGA2*‐PAs than to the polymorphous adenocarcinomas and *PLAG1*‐PAs. (B) The volcano plot shows significant differences in gene expression between *HMGA2*‐canalicular‐like PAs and CAs, including *SOX2*.

To evaluate the differences between the CAs, *HMGA2*‐canalicular‐like PAs, *HMGA2*‐PAs, *PLAG1*‐PAs, and polymorphous adenocarcinomas, a dendrogram of hierarchical clustering was constructed, which showed that the CAs were significantly closer to the *HMGA2*‐canalicular‐like PAs and *HMGA2*‐PAs than to the polymorphous adenocarcinomas and *PLAG1*‐PAs (Figure 3A).

**Figure 4:**
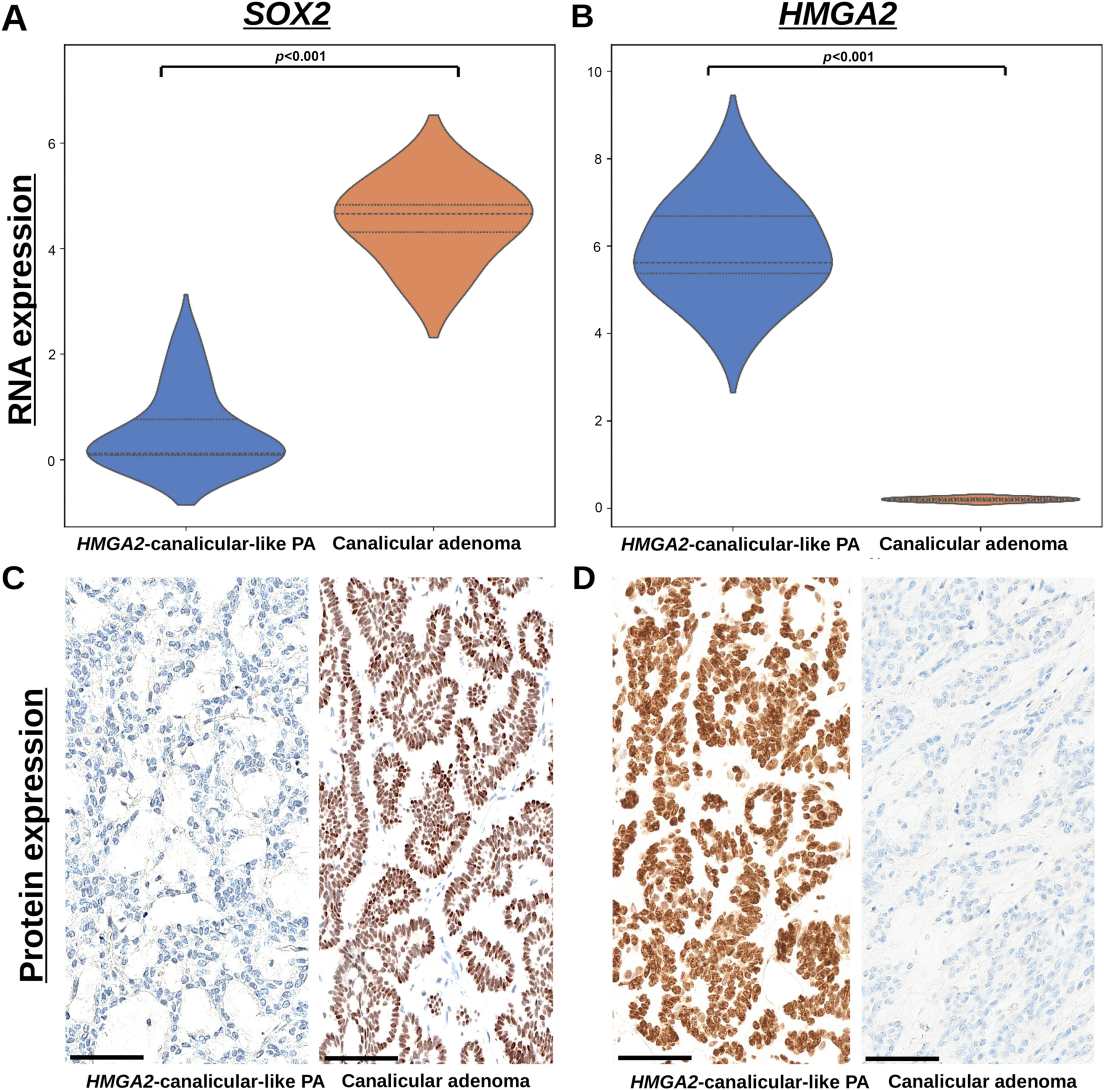
Analysis of SOX2 and HMGA2 expression between canalicular adenomas (CAs) and *HMGA2*‐canalicular‐like pleomorphic adenomas (PAs). (A, B) Violin plots illustrate significant differences in up‐ or down‐regulation of *SOX2* and *HMGA2* RNA expression between the two groups. (C, D) Immunohistochemistry illustrates a significant difference in the up‐ or down‐regulation of SOX2 and HMGA2 protein expression between the two groups (×20; scale bar, 50 μm).

**Figure 5:**
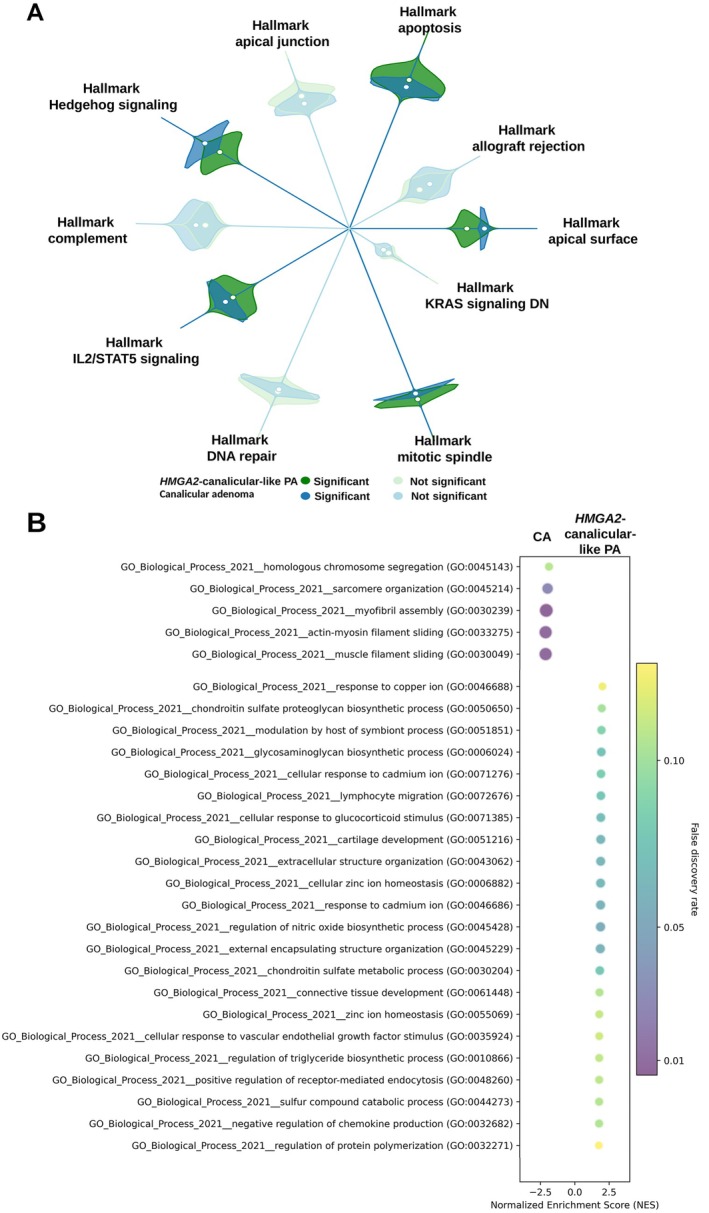
Gene set enrichment analyses between *HMGA2*‐canalicular‐like pleomorphic adenomas (PAs) and canalicular adenomas (CAs). (A) The stellar violin radar plot shows significant differences in enrichment of five hallmarks between *HMGA2*‐canalicular‐like PAs and CAs. (B) The enrichment analysis of Gene Ontology (GO) illustrates significant enrichment in CAs compared with *HMGA2*‐canalicular‐like PAs for myofibril assembly, muscle filament sliding, actin–myosin filament sliding, and sarcomere organization (*p* < 0.05).

Since the CAs and *HMGA2*‐canalicular‐like PAs shared strong morphological similarity, a volcano plot of the differential expression analysis was constructed. Five genes were significantly downregulated in the *HMGA2*‐canalicular‐like PAs compared with the CAs (FDR < 0.05: *PITX2*, *EDN3*, *DEPDC7*, *SOX2*, and *SLC37A2*) (Figure 3B). Next, the expression of SOX2 and HMGA2 was explored at the RNA and protein levels in *HMGA2*‐canalicular‐like PAs and CAs. Significantly higher expression of SOX2 was found in CAs compared with *HMGA2*‐canalicular‐like PAs at the RNA and protein levels (Figure 4A,B). Conversely, significantly greater expression of HMGA2 was found at the RNA and protein levels in *HMGA2*‐canalicular‐like PAs compared with CAs (Figure 4A–D). Analysis of the Molecular Signatures Database (MSigDB) in CAs and *HMGA2*‐canalicular‐like PAs displayed significant differences for five hallmarks. While higher enrichment was found for hallmarks of Hedgehog signaling, IL2/STAT5 signaling, and apical surface in CAs, lower enrichment was found for hallmarks of apoptosis and mitotic spindle (Figure 5A). Gene Ontology enrichment analysis displayed significant enrichment of biological process for CAs in comparison to *HMGA2*‐canalicular‐like PAs for myofibril assembly, muscle filament sliding, actin–myosin filament sliding, and sarcomere organization (Figure 5B; *p* < 0.05).

## Discussion

In the present study, CAs and *HMGA2*‐canalicular‐like PAs mostly shared similar histology and immunostaining, while the tumor location and transcriptomic profiles were different. A novel finding was the identification of significantly higher SOX2 expression among cases diagnosed as CA compared with those diagnosed as *HMGA2*‐canalicular‐like PA. The CAs displayed higher enrichment of hallmarks for Hedgehog signaling, IL2/STAT5 signaling, and apical surface, as well as lower enrichment of hallmarks for apoptosis and mitotic spindle. Additionally, Gene Ontology enrichment analysis displayed significant enrichment of biological process for CAs, compared with *HMGA2*‐canalicular‐like PAs, for myofibril assembly, muscle filament sliding, actin–myosin filament sliding, and sarcomere organization.

In this study, as elsewhere, the CAs and *HMGA2*‐canalicular‐like PAs shared numerous histological and immunohistochemical features; the primary differential marker reported was high expression of the HMGA2 protein. Of note, studies previously described that high expression of HMGA2 was found in cases harboring *HMGA2* fusion, predominantly *HMGA2*::*WIF1* [3, 4]. Our group reported recently that the *HMGA2*::*WIF1* fusion was frequently identified across all subsets of PA (pure canalicular‐like, hybrid canalicular‐like/conventional, and conventional PAs) without a predominance for any particular subset [4]. The diagnostic challenge at the histological level lies in the presence or absence of a conventional component within a tumor showing a canalicular‐like pattern. The identification of such a conventional component of PA supports the diagnosis of PA and excludes CA. In contrast, when this component is absent, additional immunohistochemical analyses, particularly the evaluation of HMGA2 and SOX2 expression, become essential. Additionally, in the present study, the *HMGA2*‐canalicular‐like PA and the conventional *HMGA2*‐PA clustered together regarding RNA expression profiles, in line with previously described studies utilizing DNA methylation analysis [10, 11], thereby supporting the absence of a strict genotype–phenotype correlation in *HMGA2*‐rearranged PAs. Conversely, we investigated whether CAs and *HMGA2*‐canalicular‐like PAs, apart from HMGA2 protein expression, would exhibit similar RNA expression profiles. The present data revealed significant transcriptomic differences, reinforcing the idea that there is no strict genotype–phenotype correlation, although this hypothesis warrants further validation.

Pathway analysis revealed a significant upregulation of muscle‐related transcriptional pathways in CAs and a trend toward downregulated chondroid transcriptional pathways in *HMGA2*‐canalicular‐like PAs. These findings suggest that the appearance of CAs may arise from distinct pathophysiological mechanisms. Interestingly, despite the upregulation of muscle pathways, CAs showed an absence of differentiated muscle lineage markers at the protein level. Moreover, the overexpression of *SOX2* and *PITX2* supports the hypothesis of a stem cell‐like phenotype, as these transcription factors are involved in the regulation of adult muscle stem cells [12, 13, 14]. Particular interest was given to *SOX2*, a potential immunohistochemical marker to distinguish CA, which has been proposed in other contexts, although its utility has not yet been fully proven [12, 13, 14, 15]. SOX2 was also identified as significantly expressed in CA, Warthin's tumor, mucoepidermoid carcinoma, and clear cell carcinoma using IHC and western blotting [15]. SOX2 protein expression has been documented in embryogenesis and more specifically in the oral cavity, contrasting with findings in major salivary glands [16]. This could further support the tumorigenesis of CAs in the oral cavity.

The *SOX2* gene, located at 3q26.33, encodes a transcription factor essential to maintain self‐renewal in undifferentiated embryonic stem cells and is also known as SRY (sex‐determining region Y‐box 2). It plays a crucial role in embryonic stem cell pluripotency and has been associated with aggressive tumor behavior [17, 18]. Other studies have shown that dysregulation of SOX2 expression plays a crucial role in tumor pathogenesis, although its effects may vary depending on the tumor type [19]. For example, SOX2 may play an important role in the carcinogenesis and progression of CXPA and is also related to prognostic indicators in CXPAs with extracapsular invasion [20]. In contrast with PA without malignant transformation and residual PA areas, SOX2 expression began at the intraductal carcinoma phase (the earliest, being retained during the progression from intraductal) to extraductal intracapsular, and finally to extracapsular carcinoma. These findings suggest that the acquisition of SOX2 expression is an early event in the carcinomatous transformation of PA [20]. SOX2 has been associated with carcinogenesis due to its role in reprogramming differentiated somatic cells into a pluripotent stem cell‐like state [21, 22, 23, 24]. This ‘stemness’ may contribute to tumor development and progression, mainly by allowing the capacities of self‐renewal and differentiation, which are important features for tumor‐initiating and tumor‐propagating cells [22]. Although SOX2 has been considered as an important antigen of stem cell‐like tumor‐initiating cells [25, 26], its roles in the carcinogenic process are still being investigated. As in CXPA, SOX2 overexpression was found in malignant transformation of precursor lesions of the pancreas, lung, cervix, and vulva [27, 28, 29, 30], but its expression was shown to be lost in the neoplastic progression of Barrett's esophagus [31]. These divergent results indicate that multiple mechanisms are involved in the regulation of SOX2 protein during the complex processes of carcinogenesis that occur in different organs, and such interactions are probably influenced by extrinsic factors and interactions in the tumor microenvironment.

The present study has limitations, including the sample size and a recruitment bias, as our center is a referral institution with specific expertise in managing complex head and neck tumors. However, even under these conditions, all cases were analyzed in depth at the transcriptomic level, revealing significant distinctions in transcriptomic and immunostaining profiles, particularly for SOX2. These findings could potentially lead to new diagnostic tools for CA identification.

## Conclusions

CAs and *HMGA2*‐canalicular‐like PAs exhibited similar histology and immunostaining but differed significantly in clinical presentation, tumor site, and transcriptomic profiles. The latter revealed significant activation of muscle‐related transcriptional pathways, and overexpression of SOX2 in CAs.

## Author contributions statement

NB conceived and designed the study. ZA and NB coordinated and managed the project. NB, BT and FT conducted the bioinformatic analyses. DP generated and analyzed the molecular data. AC, ZA and NB analyzed the histological data. PC, MF and PP collected and analyzed the clinical data. ZA and NB drafted the manuscript. All authors contributed to reviewing and editing the manuscript and approved the final version of the manuscript.

## Data Availability

The data that support the findings of this study are available from the corresponding author upon reasonable request.
